# Annotated genetic linkage maps of *Pinus pinaster* Ait. from a Central Spain population using microsatellite and gene based markers

**DOI:** 10.1186/1471-2164-13-527

**Published:** 2012-10-04

**Authors:** Marina de Miguel, Nuria de Maria, M Ángeles Guevara, Luis Diaz, Enrique Sáez-Laguna, David Sánchez-Gómez, Emilie Chancerel, Ismael Aranda, Carmen Collada, Christophe Plomion, José-Antonio Cabezas, María-Teresa Cervera

**Affiliations:** 1 INIA-CIFOR, Departamento de Ecología y Genética Forestal, Carretera de la Coruña Km7.5, Madrid, 28040, Spain; 2 Unidad Mixta de Genómica y Ecofisiología Forestal, INIA/UPM, Madrid, Spain; 3 ETSIM, Departamento de Biotecnología, Ciudad Universitaria s/n, Madrid, 28040, Spain; 4 INRA, UMR1202 BIOGECO, Cestas, F-33610, France; 5 Université de Bordeaux, UMR1202 BIOGECO, Talence, 33170, France

**Keywords:** *Pinus pinaster*, Genetic linkage map, Functional annotation, Microsatellites, SNPs

## Abstract

**Background:**

*Pinus pinaster* Ait. is a major resin producing species in Spain. Genetic linkage mapping can facilitate marker-assisted selection (MAS) through the identification of Quantitative Trait Loci and selection of allelic variants of interest in breeding populations. In this study, we report annotated genetic linkage maps for two individuals (C14 and C15) belonging to a breeding program aiming to increase resin production. We use different types of DNA markers, including last-generation molecular markers.

**Results:**

We obtained 13 and 14 linkage groups for C14 and C15 maps, respectively. A total of 211 and 215 markers were positioned on each map and estimated genome length was between 1,870 and 2,166 cM respectively, which represents near 65% of genome coverage. Comparative mapping with previously developed genetic linkage maps for *P. pinaster* based on about 60 common markers enabled aligning linkage groups to this reference map. The comparison of our annotated linkage maps and linkage maps reporting QTL information revealed 11 annotated SNPs in candidate genes that co-localized with previously reported QTLs for wood properties and water use efficiency.

**Conclusions:**

This study provides genetic linkage maps from a Spanish population that shows high levels of genetic divergence with French populations from which segregating progenies have been previously mapped. These genetic maps will be of interest to construct a reliable consensus linkage map for the species. The importance of developing functional genetic linkage maps is highlighted, especially when working with breeding populations for its future application in MAS for traits of interest.

## Background

Maritime pine (*Pinus Pinaster* Ait.) is one of the most important species in the Mediterranean region for its ecology and wood productiveness. As other conifers, this long lived species dominates different landscapes and can withstand severe environmental conditions [1]. Several studies have revealed high levels of phenotypic variation [2-4] and genetic diversity [5-7] in maritime pine. This species has a fragmented geographic distribution that could be subdivided into different meta-populations based on its high level of genetic differentiation [8-10]. In the Iberian Peninsula different patterns of local adaptation have been identified [11]. Besides its ecological value, maritime pine is also a significant species for its economic importance. Particularly, *P. pinaster* is a major resin producing species in the Iberian Peninsula [12].The resin is at the basis of many manufactured products such as turpentine, oils, varnishes, sealing wax, plastics and others. In 1990s resin tapping was reintroduced in Spain after a drastic reduction in 1970s due to the international crisis in this sector [13]. Many of the abandoned stands have been tapped again. In particular, natural stands of Central Spain are one of the most important resin tapping regions [14]. As resin production shows high heritability [15] a breeding program is a useful strategy to improve productiveness [16]. Consequently several breeding programs have been implemented for resin production in maritime pine [17-19].

Genetic linkage mapping can facilitate marker-assisted selection (MAS) as it allows the identification of quantitative trait Loci (QTL) [20-23]. Furthermore, as genome organization is well conserved in conifers, comparative mapping is a useful strategy to find homologous chromosomal segments involved in the genetic control of economical and adaptive traits [24,25].

Traditional molecular makers, such as proteins, RFLPs (Restriction Fragment Length Polymorphisms), RAPDs (Random Amplified Polymorphic DNAs), AFLPs (Amplified Fragment Length Polymorphisms) and nSSRs (nuclear Simple Sequence Repeats) have help to build a first generation genetic linkage maps in forest trees [26,27]. The use of RAPDs and AFLPs, randomly distributed in the genome [28,29], has allowed the construction of genetic linkage maps from species with large genome sizes like conifers [30-32]. An alternative for species with extremely large genomes or for populations with low levels of polymorphism are SAMPL markers (Selective Amplification of Microsatellite Polymorphic Loci). SAMPL combines the advantages of AFLPs and microsatellites resulting in higher percentage of polymorphic markers per assay and higher repeatability between assays [33].

In recent years, efforts have focused in sequencing genes of interest to build genetic linkage maps with direct functional information [34]. Functional genetic linkage maps have experienced a revolution with the availability of new sets of markers from coding regions such as: EST-Ps (Expressed Sequence Tags Polymorphisms), EST-SSRs (EST derived microsatellites) and SNPs (Single Nucleotide Polymorphisms) [35-37]. Functional genetic linkage maps based on annotated genes allow to assess redundant and paralogous EST markers and further improve the quality and utility of genetic maps [38]. Specifically, SNPs have several advantages for their use as molecular makers because they are very abundant in the genome, they show higher stability than SSRs, are usually bi-allelic and codominant [39,40]. Moreover, new technologies have been developed for high throughput detection and genotyping of SNPs reducing the cost of assays [41,42]. Thus, highly saturated genetic linkage maps can be constructed even for species with large and un-sequenced genomes like conifers [21,43-46].

As other pines, *P. pinaster* is a diploid organism characterized by a large and complex genome with high low-copy fraction [47,48]. Particularly, maritime pine has 2n = 24 chromosomes and its genome size is estimated between 51–62 pg/2C [49,50]. Several genetic linkage maps have been developed for maritime pine based on proteins [51-54], RAPDs [54-57], AFLPs [44,49,54,58], SSRs [44,58-60], EST-Ps [44,58,61] and SNPs [44]. Also, comparative mapping have been performed with *Pinus taeda* L. [44,61]. None of the genetic linkage maps available for *P. pinaster* has been derived from individuals belonging to Spanish populations*.* These populations show high levels of genetic divergence with the French populations used to design mapping progenies in previous genetic linkage maps [8]. As maritime pine shows a fragmented geographic distribution with high levels of population genetic structure and variation [6,8] it is important to explore the genetic organization in a representative population from the Castilian Plateau (Central Spain) and thus better cover the natural distribution of the species.

Thus, the main objective of this work was to construct saturated genetic linkage maps for *P. pinaster* using controlled crosses between two trees that take part in a breeding program for resin production in a natural population from Central Spain, as a first step to the genetic dissection of this trait. Combining different kind of molecular markers we aim to construct a map with annotated gene functions and homologous markers with previous maps for contributing to the development of a consensus map for the species. A second objective was to identify candidate genes overlapping with QTL already detected in this species [62-64].

## Methods

### Mapping populations

Two outbred full-sibs families of *P. pinaster* were used for genetic linkage mapping. Progenies were originated from two reciprocal controlled crosses between two progenitors (C14 and C15) belonging to a natural population in Coca (Segovia) located in Central Spain (41° 12’ N 4° 31’ W). Previous studies on this population have showed a differential genetic structure when compared with other populations of the natural distribution of the species [8]. Progenitors took part in a breeding program for resin production started in 1994 and they were selected for their contrasting resin production, low for C14 and higher for C15. Controlled crosses were carried out in 1999 for C14xC15 and in 2000 for C15xC14. F_1_ seeds were collected and germinated in controlled conditions at Instituto Nacional de Investigación y Tecnología Agraria y Alimentaria, INIA (Madrid, Spain). Then they were planted in semi-controlled conditions at Dirección Nacional de Biodiversidad– Madrid (40° 27’ N 3° 44’ W). A paternity test analysis was performed with 13 SSRs. Finally, once the contaminants were removed, the mapping population comprised 161 individuals: 106 from family C14xC15 and 55 individuals from C15xC14.

### Molecular markers

Genomic DNA was extracted from needles using a modified protocol from Dellaporta et al. [65] for all marker analyses, but for the 1536 and 384 GoldenGate assays (Illumina Inc., San Diego, CA, USA), for which a commercial *Invisorb DNA plants HTS96kit* (Invitek GmbH, Berlin, Germany) was used. Four types of molecular marker were used for genotyping the mapping populations: nSSRs, EST-Ps, SAMPLs and SNPs.

nSSRs: Forty seven primer pairs designed for amplification of nSSR loci in *P. pinaster* and *P. taeda*[60,66,67] were tested for segregation in the mapping populations. Thirteen loci were polymorphic, 27 were monomorphic, and seven resulted in muti-banding or non-clear patterns. Amplification of A6F03, A5B01, A5A11, A6F10, A6D04, A5B07 loci was performed as in Guevara et al. [66]. Amplification of NZPR823, NZPR413,NZPR114, NZPR544, SsrPt_ctg64, SsrPt_ctg275 loci was performed as described by Chagné et al. [60] and the amplification of PtTX3116 followed the protocol described by Auckland et al. [68] with modified touchdown profile, using 55°C and 45°C as starting and final temperatures [69]. A Perkin-Elmer GenAmp 9700 thermal cycler (Perkin Elmer Inc., Waltham, Massachusetts, USA) was used to carry out PCR reactions. Amplified products were separated in denaturing gels containing 6% acrylamide / bisacrylamide (19:3), 7 M urea and 1x TBE. Amplified products were visualized in a DNA Analyzer System (4300, LI-COR Biosciences, Lincoln, NE, USA). Fragments were scored visually as codominant markers.

EST-Ps: EST-P genotyping was carried out by Tilling (Targeting Induced Local Lesions in Genomes) as described by Till et al. [70]. This technique allows detection of multiple SNP sites heterozygous in the same progenitor [71]. A set of 14 EST-P primer pairs (PtIFG_893, PtIFG_9136, PtIFG_9034, PtIFG_1955, PtIFG_8429, PtIFG_8702, PtIFG_3C8E, PtIFG_22B8, PtIFG_1CA6C, PtIFG_9044, PtIFG_2253, PtIFG_8436, PtIFG_8887, PtIFG_C6H11) derived from cDNA sequences of *P. taeda* and *P. pinaster*[61,72,73] were tested, in order to identify the most informative markers. A total of 11 EST-P primer pairs generated 25 polymorphic markers. PCRs were performed in 10 μl containing 10 ng of DNA; 1x PCR reaction buffer (Fermentas, Ontario, Canada), 0.2 mM of each dNTP, 2 mM MgSO_4_, 0.25U Pfu DNA polymerase (Fermentas, Ontario, Canada), 0.2 μM of each primer (forward primers were labeled on its 5’ end with IRDye 700 and reverse primers with IRDye 800). A Perkin-Elmer GenAmp 9700 thermal cycler (Perkin Elmer Inc., Waltham, Massachusetts, USA) was used to carry out PCR reactions. Thermocycler parameters were: 94°C 2 min, 10 touchdown cycles of 94°C 20s, (Tm + 3)°C, 45 s (−0.8°C/cycle), 72°C 1 min; 45 cycles of 94°C 20s, (Tm-5)°C 45 s, 72°C 1 min and final extension step of 72°C for 7 min. Amplification products were visualized on 1% agarose gels to verify amplification. PCR products were digested with CEL I nuclease purified as described by Till et al. [8]. Previously, the concentration of nuclease added, was screened to optimize the detection of heteroduplex between heterozygous sites. Partial DNA digestions were stopped by the addition of 5 μl of 0.5 M EDTA. The mixture were transferred to 96-well Sephadex G50 spin plates (GE HeathCare, Waukesha, WI, USA) for cleaning up by centrifugation into formamide solution and heated at 70°C to reduce the volume to 8 μl. DNA fragments were separated in denaturing gels containing 8% Long Ranger polyacrylamide (Cambrex, East Rutherford, NJ, USA), 7 M urea and 1x TBE. Fragments detection was carried out on a DNA Analyzer System (4300, LI-COR Biosciences, Lincoln, NE, USA). Fragments were scored as dominant markers. Polymorphism was inferred from the resulting fragment pattern and confirmed by sequencing independently undigested amplified products from four haploid megagametophyte DNAs for each progenitor.

SAMPLs: SAMPL genotyping was performed as indicated by Vos et al. [28] with several modifications [74]. Preamplifications were carried out using three primer combinations (*Eco*RI + A/ *Mse*I + G; *Eco*RI + A/ *Mse*I + C; *Eco*RI + A/ *Mse*I + T). For the selective amplification a SAMPL primer [CATA: (CA)_8_(TA)_2_; GATA: (GA)_8_(TA)_2_[75]], was used in combination with an *Eco*RI + 3 primer. In order to select the most informative combinations (those with a higher level of polymorphism) different combinations were tested using template DNA from the parental lines and 9 offspring. Progenitor C14 revealed lower levels of polymorphisms than C15 (see *Results* section), thus primer combinations were chosen in order to equilibrate the number of markers segregating from each progenitor. A total of 31 CATA*/Eco*RI and 26 GATA*/Eco*RI primer combinations were used for the selective amplification. Selective PCR reaction were performed in 10 μl of 1x PCR Buffer (10 mM Tris–HCl, 50 mM KCl, pH 8.3), 0.1 mM of each dNTP, 2.5 mM MgCl_2_ (Roche, Basel, Switzerland), 3 ng IRDye 800 5’end labeled CATA or GATA primers, 15 ng *Eco*RI + 3 primer, 0.2U Taq DNA polymerase (Invitrogen, Grand Island, NY, USA) and 5 μl of 10-fold diluted pre-amplification DNA fragments using classical AFLP cycling parameters [12]. Samples were loaded into denaturing gels containing 8% Long Ranger polyacrylamide (Cambrex, East Rutherford, NJ, USA), 7 M urea and 1x TBE. Fragments detection was carried out on a DNA Analyzer System (4300, LI-COR Biosciences, Lincoln, NE, USA). Fragments were scored visually as dominant markers.

SNPs: two SNP genotyping assays were used in this study; a 1,536 BeadArray™ and a 384 BeadXpress® Golden Gate assays (Illumina Inc., San Diego, CA, USA). SNPs selected for 1,536 Golden Gate assay corresponded to three different sets (see Chancerel et al. [44] for further details): *in vitro* polymorphisms from 35 candidate genes for cell wall formation and drought stress resistance; *in silico* SNPs from a maritime pine EST assembly; and *in silico* polymorphism from re-sequenced amplicons of the species. In this genotyping assay, 95 DNA samples of the mapping progenies were genotyped (73 for C14xC15 and 22 for C15xC14). In order to increase the number of genotyped individuals for a set of genes of interest, another genotyping assay was developed. This genotyping assay (384 SNPlex) consisted in a subsample of SNPs selected from the 1,536 genotyping assay and 14 additional SNPs from candidate genes for drought resistance [76]. It was carried out at Center for Genomic Regulation (CRG, Barcelona, Spain) for a total of 119 DNA samples (79 for C14x15 and 40 for C15xC14). Both genotyping assays were realized according to the manufacturer’s instructions (Illumina Inc., San Diego, CA, USA) and SNPs clusters revised manually with Illumina Bead Studio v2.0 Software. When the same SNP was successfully genotyped in both assays priority was given for the 384 Vera Code data because of the higher number of DNA samples genotyped in this assay. Contig and gene sequences containing the polymorphic SNPs are presented in Additional file 1.

### Linkage map construction

For each progenitor we assembled three different linkage maps belonging to datasets of C14xC15 (106 individuals), C15xC14 (55 individuals) and a dataset with the information of the individuals of both reciprocal crosses (161 individuals). Since no relevant differences were found as a consequence of merging both progenies (see *Results* section), further linkage analyses were developed using only the data set with the merged information of both progenies. Parental maps were constructed using the “two-way-pseudo-testcross” mapping strategy [77]. Markers with more than 70% of missing data were excluded from further analysis. Linkage analyses and map estimations were performed using the regression mapping algorithm implemented in the software JoinMap v4.0 [78] with the CP population type and using a recombination fraction < 0.35 and a LOD > 3 as mapping parameters. Map distances were calculated using Kosambi mapping function [79]. When difficulties in estimating marker order are found, two additional maps are constructed (map2 and map3). In map2, new markers are added because more pairwise data are available. In map3, the remaining loci are added by decreasing statistical support. In these cases we kept map2 for further analyses. When a pair of markers was considered identical, only one of the markers was selected for mapping. In order to assign unlinked loci to selected linkage groups (LG), the strongest cross link was employed with a LOD value of 3 (JoinMap command “assign ungrouped loci to SCL-groups”). Segregation ratios were tested using χ^2^ test (P ≤ 0.01).

### Evaluation of homogeneity of recombination rate between female and male meiosis

In order to evaluate whether the male and female gametes presented different levels of recombination, we tested departure from homogeneity of recombination fraction following Plomion et al. [57]. Since the statistical power of homogeneity depends largely on the sample size, the test was performed for all markers pairs in common in the three genetic maps for each progenitor and having a recombination fraction lower than 0.1 (Additional file 2).

### Comparative mapping

The linkage maps of both progenitors were compared based on common markers. Besides genetic maps were compared with previously developed *P. pinaster* maps [44] based on common SSRs, ESTPs and SNPs. LGs were named according to Chancerel et al. [44] using loblolly pine nomenclature, as it is the reference pine species.

### Genome length and map coverage

Total genome length was calculated as the sum of all mapped marker intervals. Estimated genome length (*G*_*e*_), was determined from the partial linkage data according to Hulbert et al. [80] modified by Chakravarti et al. [81] (Method 3). A minimum LOD score of three was chosen to estimate genome length using framework maps constructed following the methodology previously described in order to avoid overestimation of genome size because of clustered markers. Observed map coverage was calculated as the ratio of total genome length to estimated genome length [82].

### Marker distribution

To evaluate whether markers were randomly distributed, we tested the procedure explained in Echt et al. [38]. A Kolmogorov-Smirnov test for two populations was implemented to compare the observed marker distribution frequencies with expected distribution frequencies under the assumption of randomness. SAMPLs and SNPs distribution were also analyzed by calculating Pearson correlation coefficient between the number of SAMPLs and SNPs in the LGs and the size of the LGs as in Cervera et al. [82].

### Heterozygosity levels

The average heterozygosity was estimated for each progenitor and for each molecular marker type independently. Heterozygosity levels based on SSRs were calculated as the ratio between polymorphic and total number of tested SSRs, discarding those with multibanding and non-clear patterns. Three SSR primer pairs resulted in the amplification of two different loci with clearly different segregation patterns and were scored as different markers, but they were considered as only one for heterozygosity estimations. Heterozygosity levels based on SNPs were calculated as the ratio of polymorphic SNPs and total number of SNPs successfully genotyped. Heterozygosity estimates for SAMPLs were calculated for the first primer combination tested; since the following ones were selected in order to maximize the number of polymorphic markers in C14 (see *Molecular markers* subsection). Heterozygosity based on EST-Ps was not calculated because we only analyzed markers that had been found polymorphic in previous studies in other pine species [61,72,73], therefore a bias could be introduced.

### Functional annotation

Functional annotation of the mapped SNP-based genes was carried out using sequence information from the Oligo Pool Assay-OPA (60 nucleotides in length at both sides of the SNP position). In order to obtain homology with longer sequences a BLAST-N search was performed using the pine Gene Index [83] and GeneBank [84]. We retained sequences showing the highest homology (e-value lower than 10^-20^ were considered significant). Then, these longer sequences were annotated using Blast2GO software [85]. Gene Ontology (GO) annotation terms for molecular function at ontology level equal to 3 were placed in the map in order to search for clusters of genes with similar function. For sequences where GO annotation for level 3 was not available we selected the GO annotation terms for level 2. To evaluate whether similar GO terms were clustered or randomly distributed along the genome we performed for each GO term at level 2 a Kolmogorov-Smirnov test for two populations as explained in *Marker Distribution* subsection.

In order to detect interesting co-localizations between candidate genes and QTLs the linkage maps developed in this study were aligned with maps previously constructed for *P. pinaster* containing enough number of orthologous markers to detect homologous LGs and the respective position of QTLs for different traits [44,61,86].

### Marker nomenclature

Marker nomenclature for SSRs and SNPs were maintained according to their original publications (see *Molecular Markers* subsection). EST-Ps also conserved original nomenclature, but the size of the amplified band was added to the marker name. SAMPLs were named with the differential selective nucleotide used in the preamplification (C, G or T), followed by the targeted microsatellite (CATA or GATA), and the selective *Eco*RI + 3 primer employed, ending by the size of the amplified band fragment.

## Results and discussion

The paternity test analysis revealed seven contaminants for C14xC15 and three for C15xC14 that were removed for further analyses. The final number of individuals per progeny, 106 for C14xC15 and 55 for C15xC14, was in the limit for reliable estimations and as we did not observe significant differences in recombination fraction between female and male meiosis (see next subsection) we constructed the genetic linkage maps by pooling all individuals of both reciprocal crosses.

### Evaluation of homogeneity of recombination between female and male meiosis

Ninety-six marker pairs for C14 and 42 for C15, with a recombination fraction below 0.1, were available in all three maps (C14xC15, C15xC14 and pool map) (see *Methods* section). Eight marker pairs out of the 96 analyzed, showed significant differences between female and male meiosis for C14 (data not shown). Five of them showed a higher recombination rate for male meiosis and three for female meiosis. No marker pair resulted in significant differences in recombination rate for C15. The low level of differences detected in recombination fraction between female and male meiosis supports the merging of both progenies in order to obtain a higher number of offspring in the mapping population and thereby establishes more precise parental maps. No evidence of heterogeneity of recombination was previously reported for *P. pinaster*[56] and other conifer species [87]. However, Plomion and O’Malley [57] suggested that recombination fraction could be higher in male meiosis for *P. pinaster*. The important differences in number of individuals between our mapping progenies (106 versus 55) compelled us to perform the analyses with a narrow window of markers (only those with a recombination fraction lower than 0.1), while in Plomion and O’Malley [57] analyses were performed with a wider window (markers pairs with a recombination fraction lower than 0.3). This could explain the difference in results obtained. Nevertheless, further research in testing homogeneity of recombination between female and male meiosis is needed to clarify whether or not female and male gametes exhibit similar recombination rate, which can have some implications for MAS.

### Individual linkage maps and comparative mapping

Previous analysis of the mapping population with four AFLP primer combinations revealed very low levels of polymorphism (data not shown). Therefore, we decided to use SAMPL technique to increase the number of polymorphic fragments. SAMPL analysis was performed using the most informative primer combinations (see *Methods* section). This result validates the use of SAMPLs as an alternative for genotyping low polymorphic populations.

Out of the total set of molecular markers available (Table 1), four and five markers were excluded from C14 and C15 datasets respectively, because of their identical segregation profiles with other markers. All of them were SNPs belonging to the same gene or contig. Markers with more than 70% of missing data were also excluded. Most of them were SAMPLs genotyped only in the C15xC14 pedigree. In addition, in a “two-way-pseudo-test-cross” mapping strategy, intercross markers i.e. markers with the same heterozygous allelic configuration in both progenitors, are less informative. Because of that, several SAMPLs, SNPs and one microsatellite marker were excluded. However, when it was possible we kept a number of intercross markers because they allow to align homologous LGs between both parental maps.

**Table 1:** Mapping parameters of parental linkage maps constructed by merging two reciprocal crosses: C14xC15 and C15xC14

**Mapping parameter**	**C14**	**C15**
Total number of available markers	402	410
Number of SSRs loci	11	13
Number of ESTP loci ^a^	13	12
Number of SAMPL loci	228	237
Number of SNP loci ^b^	150	148
Total number of distorted (p ≤ 0.01) markers	39	52
Number of excluded markers ^c^	62	72
Number of SSRs loci	1	1
Number of ESTP loci	0	0
Number of SAMPL loci	46	60
Number of SNP loci	15	11
Number of markers not excluded	340	338
Number of assigned markers ^d^	321	319
Number of SSRs loci	9	10
Number of ESTP loci	11	11
Number of SAMPL loci	174	166
Number of SNP loci	127	132
Number of positioned markers ^e^	215 (63.2%)	211 (62.4%)
Number of SSR loci	6 (60%)	7 (58.3%)
Number of ESTP loci	10 (76.9%)	7 (58.3%)
Number of SAMPL loci	98 (53.8%)	98 (55.4%)
Number of SNP loci	101 (74.8%)	99 (72.3%)
Number of distorted (p ≤ 0.01) positioned markers	14	14
Unlinked markers (%) ^f^	19(6.3%)	19 (5.6%)
Number of LG >3 before making alignments	22	20
Number of LG >3 after making alignments	13	14
Smallest LG (cM) before making alignments	17.5	13.4
Largest LG (cM) before making alignments	81.1	155.3
Average length (cM) LG ± SD before alignments	53.7 ± 20.6	69 ± 35.6
Smallest LG (cM) after making alignments	52	42.3
Largest LG (cM) after making alignments	142.2	155.3
Average length (cM) of a LG ± SD after alignments	90.8 ± 29.14	98.5 ± 38
Maximum distance (cM) between 2 adjacent markers	24.9	35.3
Average distance (cM) between 2 adjacent markers ± SD	6.12 ± 5.8	7.22 ± 6.4
Observed map length (cM)	1180.4	1379.5
Estimated map length (cM)	1870.2	2166.6
Observed map coverage	63%	64%

^a^ The 25 ESTP-s correspond to 11 gene loci.

^b^ The SNPs markers correspond to 47 gene loci and 143 contigs.

^c^ Markers with more than 70% of missing data (see *Methods* section) and identical markers.

^d^ Assigned markers correspond to markers linked with more than 2 other markers.

^e^ Unpositioned markers correspond to markers with a recombination frequency higher than 0.35 with the nearest linked marker (unlinked markers) or markers which position could not be reliably estimated. Percentage of positioned markers was calculated over the number of not excluded markers.

^f^ Percentage of unlinked markers was calculated over the number of not excluded markers.

*SD* Standard deviation.

**Figure 1:**
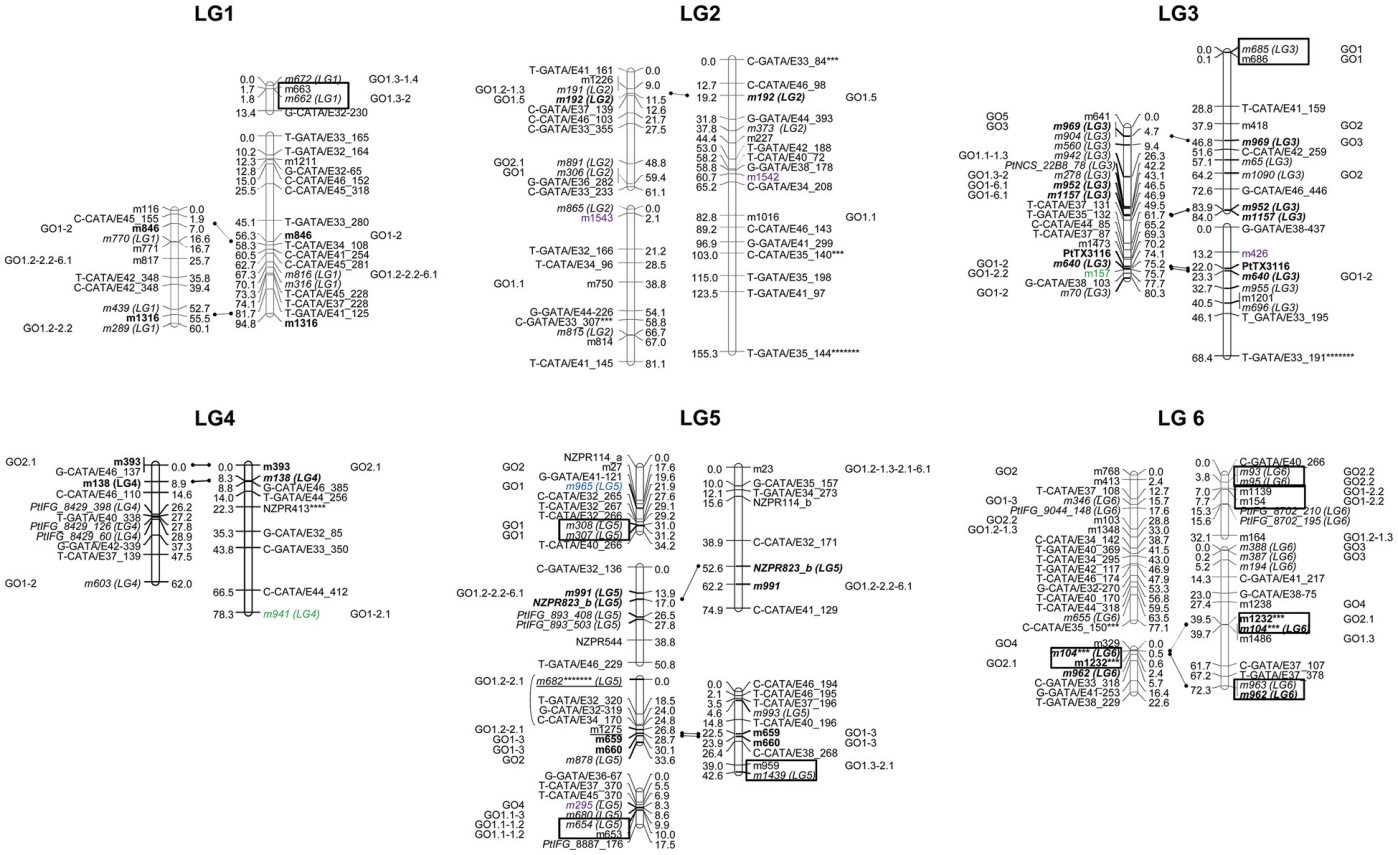
**Genetic linkage maps: LGs 1 to 6.** Bars on the left represent the LGs obtained for C14 and the bars on the right the LGs obtained for C15. Common markers between both maps are in bold and connected with a solid line. Markers in italics are in common with maps of Chancerel et al. [44] and the homologous LG in this study is indicated with brackets. Markers showing any special feature (see *Results* section) are underlined. Markers in color are candidate genes that co-localize with QTLs reported in previously published maps for wood properties (green), isotopic composition of C^13^ (violet) and ring growth (blue). SNPs belonging to the same contig are surrounded by a solid line and when they were too far from each other they are connected by a solid line in the left of the chromosome bar. Markers showing significant distorted segregation ratios are indicated with asterisks (*** means significant at 0.01 p-value, **** at 0.005, ***** at 0.001, ****** at 0.0005 and ******* at 0.0001). Annotations of SNPs are indicated by the term GO and a numeric code. Numeric codes for molecular function annotation level 2: 1 - binding; 2 - catalytic activity; 3 - structural molecule activity; 4 - transporter activity; 5 - enzyme regulator activity. Numeric codes for molecular function level 3: 1.1 - nucleic acid binding; 1.2 - nucleotide binding; 1.3 - protein binding; 1.4 - carbohydrate binding; 1.5 - lipid binding; 2.1 - hydrolase activity; 2.2 - transferase activity: 5.1 - sequence specific DNA binding; 6.1 - signal transducer activity.

**Figure 2:**
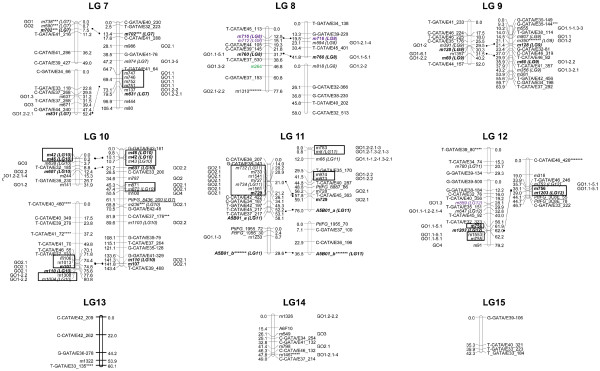
**Genetic linkage maps: LGs 7 to 15.** Bars on the left represent the LGs obtained for C14 and the bars on the right the LGs obtained for C15. Common markers between both maps are in bold and connected with a solid line. Markers in italics are in common with maps of Chancerel et al. [44] and the homologous LG in this study is indicated with brackets. Markers showing any special feature (see *Results* section) are underlined. Markers in color are candidate genes that co-localize with QTLs reported in previously published maps for wood properties (green), isotopic composition of C^13^ (violet) and ring growth (blue). SNPs belonging to the same contig are surrounded by a solid line and when they were too far from each other they are connected by a solid line in the left of the chromosome bar. Markers showing significant distorted segregation ratios are indicated with asterisks (*** means significant at 0.01 p-value, **** at 0.005, ***** at 0.001, ****** at 0.0005 and ******* at 0.0001). Annotations of SNPs are indicated by the term GO and a numeric code. Numeric codes for molecular function annotation level 2: 1 - binding; 2 - catalytic activity; 3 - structural molecule activity; 4 - transporter activity; 5 - enzyme regulator activity. Numeric codes for molecular function level 3: 1.1 - nucleic acid binding; 1.2 - nucleotide binding; 1.3 - protein binding; 1.4 - carbohydrate binding; 1.5 - lipid binding; 2.1 - hydrolase activity; 2.2 - transferase activity: 5.1 - sequence specific DNA binding; 6.1 - signal transducer activity.

Near 5% of markers used for linkage analysis were unlinked, which is in the same range of what has been observed in other conifer maps [24,45]. Most of them presented more than 35% of missing data and corresponded to SAMPLs genotyped only in the C14xC15 pedigree. Several SNPs were also unlinked. Near 94% of the markers could be assigned to LGs and 60% could be positioned in the final maps (Table 1, Figure 1, Figure 2 and Additional file 3). The lower percentage of markers positioned when compared with other highly saturated maps [46] is due to the use of SAMPLs only scoring in one of the mapping progenies, as revealed by the low percentage of positioned SAMPLs (Table 1). When we discard SAMPLs scored only in one or the two mapping progenies, the percentage of SAMPL markers positioned in the parental maps increases to 65.3% for C14 and 72.1% for C15. These results are very similar to those obtained with positioned SNPs (Table 1) indicating that both type of markers are suitable for the construction of linkage maps. Even more, for a complete coverage of the genome it is interesting to use markers with different target sequences, since coding and non-coding regions seems not to be randomly distributed along the genome [87,88].

In a first phase, before aligning on the reference *P. pinaster* linkage map, 22 LGs were obtained for C14 and 20 for C15 (Table 1, Figure 1 and Figure 2). The smallest LGs were similar in size between C14 and C15 maps. However, the largest LG were higher in C15 than in C14. Also, average size of LGs was slightly higher for C15 than for C14. This was explained because average distance and maximum distance between two adjacent markers was larger in C15 than in C14. Thirty-one intercross markers between both parental maps allowed the identification of homologous LGs (Table 2). Eleven markers with segregation 1:2:1 (same heterozygous combination in both parents) could only be positioned in one parental map (Table 2). Five of them could not be mapped in the other parent because they were ungrouped and the remaining six markers because of the increase in the goodness of fit calculated for the order of markers when were included in the map.

**Table 2:** Markers used for comparative mapping within the species

**Marker**	**C14**	**C15**
Common markers between both parental maps		33
Markers segregating in both parents positioned only in one parental map	5	6
Common markers with Chancerel et al. [44]	65	57
Common SSR loci	2	3
Common ESTP loci	7	3
Common SNP loci	56	51
Number of LGs without common markers with Chancerel et al. [44]	1	2

The alignment with maps described by Chancerel et al. [44], based on common SSRs, EST-Ps and SNPs (Table 2), made it possible to bring together some LGs resulting into 13 LGs for C14 and 14 LGs for C15 (Table 1), close to the 12 chromosomes of the haploid *P. pinaster* genome [89]. In general, similar size of LGs for parental maps was obtained except for LGs 1, 3 and 10 that were larger in C15 map and LG12 that was larger in C14 map. The fact that we could not assemble the markers in 12 LGs, the differences in size of homologous LGs and the presence of common markers only positioned in one parental map, are probably related with the presence of homozygous regions in the genome of these individuals that prevent mapping markers in these areas. This effect was partially expected because previous studies of the population of origin of both parental trees, Coca, revealed a high coefficient of endogamy [90]. As a result of endogamy we would expect a loss of polymorphisms in the individuals coming from this population, strictly confirmed by the low levels of polymorphism detected by AFLPs genotyping (data not shown) and the low levels of heterozygosity found in the parental trees (Table 3) compared with observed heterozygosity in other provenances of *P. pinaster*[5].

**Table 3:** Heterozygosity

**Marker**	**C14**			**C15**		
	**Poly.**	**Mono.**	**Heterozygosity (%)**	**Poly.**	**Mono.**	**Heterozygosity (%)**
SSR	9	31	0.23	10	30	0.25
SAMPL	133	191	0.41	251	191	0.57
SNP	150	672	0.18	96	726	0.12

Percentage of heterozygosity calculated as the ratio of polymorphic markers segregating in each parental map over total (polymorphic –Poly.- and monomorphic-Mono.-) markers.

Heterozygosity estimates for SAMPLs were calculated for the first primer combination tested in the family.

In this respect, it is important to point out to the difference in heterozygosity between C14 and C15 parental trees as revealed by the estimation obtained from SAMPLs (Table 3). This difference was overcome by further genotyping using selected SAMPL primer combinations with a higher number of polymorphic markers in C14 (see *Methods* section). Percentage of heterozygosity calculated from SSRs and SNPs yielded lower values than those obtained from SAMPLs and differences in heterozygosity between C14 and C15 could not be appreciated. One possible explanation is that analyzed SNPs were selected from coding regions where the level of polymorphism is lower than in non coding regions [91].

Twenty four contigs with several SNPs (from two to seven) were included in the linkage maps. SNPs belonging to the same gene or contig mapped always in the same position or less than 3 cM away (Figure 1 and Figure 2), except *m682* and *m127* (LG 5), separated by 26.8 cM. Marker *m682* was distorted at the 0.1% significance level, which could affect the accurateness of its position. Alternatively, both SNPs may be associated to different loci at the same LG. The fact that nearly all SNPs belonging to the same contig were mapped in the same position supports the accuracy of the genotyping method used, as previously reported [41,44].

Alignments with the linkage maps developed by Chancerel et al. [44] pointed out that marker order was highly conserved, excepting small inversions of less than 5 cM (data not shown). The only major inconsistency in data was found for marker PtIFG_8436_200, which was amplified using the same primer combination as in Chancerel et al. [44], but subjected to different detection techniques, tilling versus SSCP (Figure 2). This EST-P marker was mapped in LG 10 in our mapping progeny in agreement with previous developed maps in *P. taeda*[72]. However, in other published linkage maps of *P. pinaster* this gene was mapped in LG 7 [44,61]. Chagné et al. [61] discussed the possibility that PtIFG_8436 in *P. pinaster* targeted a paralogous gene as they found low similarity at the DNA sequence level. Our result suggests the existence of an orthologous sequence between *P. pinaster* and *P. taeda* genomes for the region amplified by PtIFG_8436 marker in LG 10 and a paralogous sequence in LG 7 of *P. pinaster* genome.

### Segregation distortion

A χ^2^ test (d.f. = 1) was performed to test Mendelian segregation of each marker. We detected 9.7% of markers showing distorted segregation ratios at 1% significance level for C14 and 12% for C15 linkage maps (Table 1). These results are very similar to those obtained in other pine [92,93] and conifer species [36]. The number of distorted markers excluded and unlinked was similar to the number of distorted markers finally assigned to LGs (Additional file 4). Besides, among the unlinked and excluded loci the number of distorted markers was not higher than those showing no segregation distortion (Table 1). Thus, in this case, unlinked and excluded loci seem not to be the result of segregation distortion, as previously reported in other linkage studies [24]. Distorted markers assigned to a LG were randomly distributed (Additional file 4). Only 13 distorted markers could be positioned in each map indicating the difficulty to estimate an accurate position for these distorted markers. Distorted markers positioned in the maps did not appear clustered in specific regions of the genome (Table 1, Figure 1 and Figure 2) suggesting that segregation distortion was probably related with genotyping errors rather than the effect of pre or post-zygotic selection. As they were not clustered they did not compromise map structure [93].

### Marker distribution

Markers were randomly distributed along the genome as no significant differences were found between distribution of markers along the LGs and expected distribution under the hypothesis of randomness (Kolmogorov-Smirnov for two populations, D = 0.55, p-value = 0.124 for C14 map and D = 0.5, p-value = 0.474 for C15 map), in accordance to other conifer maps [24,32]. Besides, largest LG had more SNPs (Pearson correlation, r =0.53, p-value = 0.01 for C14 map and r =0.62, p-value = 0.003 for C15 map) than smaller LG. Same results were obtained for SAMPLs (Pearson correlation, r =0.69, p-value < 0.001 for C14 map and r =0.75, p-value < 0.001 for C15 map) indicating that they are also randomly distributed along the genome, as expected for this kind of multiband markers [28].

### Genome length and map coverage

Observed genome length ranged from 1,180.4 (C14) to 1,379.5 cM (C15), 200 cM larger for C15 map than for C14 map (Table 1). In other *P. pinaster* maps observed genome length ranged from 869 to 1,860 cM depending on the density of markers [44,55,58]. The higher genome length observed in C15 map agrees with its higher heterozygosity estimation compared to C14 map (Table 3). Estimated genome length ranged from 1,870.2 to 2,166.6 cM, in line with what has been obtained in previous *P. pinaster* maps (1,223 to 3,252 cM depending on the method of estimation [44,56,88]). The last generation maps estimated *P. pinaster* genome size to be 2,500 cM [44], a value which is near our estimates and close to other pine species [94]. Estimated genome length was higher for C15 linkage map although the observed map coverage (near 65%, Table 1) was similar for both parental maps. High density genetic linkage maps usually report map coverage over 90% [46,95]. However, previously published *P. pinaster* genetic linkage maps also reported map coverage near 65% [44,57], indicating the difficulty to achieve a complete coverage for such a large and complex genome.

### Functional annotation

We validated and improved the functional annotation information for mapped SNPs. Significant sequence homology was found in pine Gene Index database for 160 out of the 171 mapped SNPs in both parental linkage maps [83] (Additional file 5). Sequence homology was found for several species with the top hit homologies for *Picea sitchensis*, *Pinus taeda*, *Pinus radiata*, *Vitis vinifera* and *Picea glauca* (Additional file 5). Nine sequences over the 171 sequences showed no match with the InterPro database [96] and we did not find any GO term for seven sequences. Thus, a total of 144 sequences were annotated, 132 of them for molecular function and 101 sequences with annotation for molecular function levels 2 or 3. Most of the mapped SNPs were associated to cDNA belonging to the GO terms: *binding*, *catalytic activity* and *hydrolase activity* (Additional file 6). As expected, SNPs belonging to the same contig reported identical GO annotation terms. However, our results could not confirm statistically (Kolmogorov-Smirnov test for two populations not significant, data not shown) if neighboring SNPs belonging to different genes or contigs exhibited GO terms for the same molecular function. Denser genetic maps with deeper functional annotation are required to evaluate if genes with similar functions are clustered or not.

The comparison of our annotated linkage maps and linkage maps reporting QTL information revealed candidate genes for several QTLs for wood properties or isotopic composition of C^13^ (δC^13^) [61,62,64]. δC^13^ is a character closely related with water use efficiency [97]. In our study, SNPs annotated for water-stress inducible proteins, AQUAPORINs and DEHYDRINs were positioned in the same region as QTLs for δC^13^[62] (Table 4). This outcome reinforces the hypothesis that the genomic regions identified by QTL analysis [62] might play a key role in the genetic control of water use efficiency. Also SNPs associated with a *CELLULOSE SYNTHASE CESA3*, a *PEROXIDASE* (enzyme involved in lignin polymerization [98]) and a *ENDO- 1,4BETA-XYLANASE A-LIKE* gene (Additional file 5) co-localized with QTLs for wood chemical composition and fiber properties [64] (Table 4). This result increases the evidence of function assigned to these genes and has special relevance when we consider that orthologous QTLs for wood properties were also found in other *Pinus* species [61]. This finding highlights the importance of developing functional genetic linkage maps to be used as useful tools to look for favorable allelic variants to be implemented in MAS.

**Table 4:** **Co-localizations of SNPs andQTLs**

**SNP ID**	**Sequence description**	**e-value**	**LG**	**Trait QTL**	**Reference**
m157	CELLULOSE SYNTHASE	1.5e^-68^	3	wood	Pot et al. [64]
m264	PEROXIDASE	2.9^e-147^	8	wood	
m941	ENDO- BETA-XYLANASE A-LIKE	0	4	wood	
m1542	WATER-STRESS INDUCIBLE PROTEIN 1	9.5^e-26^	2	δC^13^	Brendel et al. [62]
m1543	WATER-STRESS INDUCIBLE PROTEIN 3	7.3^e-34^	2	δC^13^	
m426	WATER DEFICIT INDUCIBLE LP3-LIKE PROTEIN	3.2^e-62^	3	δC^13^	
m295	AQUAPORIN	9.4^e-3^	5	δC^13^	
m965	THYLAKOID LUMENAL 19 KDA PROTEIN	7.1^e-119^	5	ring growth	
m712	DEHYDRIN 9 PROTEIN	1.2^e-71^	8	δC^13^	
m716	DEHYDRIN 2	1.9^e-43^	8	δC^13^	
m859	THYLAKOID LUMENAL PROTEIN	9.8^e-145^	12	δC^13^	

Co-localization of mapped SNPs with QTLs detected in previously published maps of *P.pinaster*. δC^13^ stands for isotopic composition of C^13^. *Wood* stands for wood chemical composition and fibre properties.

## Conclusions

Our study demonstrates the importance of developing genetic linkage maps from different populations representing different genetic backgrounds in order to generate an accurate consensus linkage map of the same species. Comparative mapping is a key process to facilitate the understanding of genome organization and evolution in conifers. For that purpose it is essential to correctly identify orthologous versus paralogous genes. New efforts in detecting orthologous markers as well as progress in sequencing conifer genomes will improve comparative mapping studies in the future. Here we also confirm the importance of developing functional genetic linkage maps, especially when working with breeding populations for its future application in MAS for traits of interest.

## Competing interests

The authors declare that they have no competing interests.

## Authors’ contributions

MdM: Analyzed the quality of SNP genotyping results, constructed the linkage maps and wrote the first draft of the manuscript. NdM, MAG and LD: Carried out SSRs, EST-Ps and SAMPL analyses. DSG: Handled and managed plant material. EC and CP: helped MdM in analyzing SNPs from de 1,536 SNP array. ESL: Carried out DNA extraction. CC: Performed functional annotation. JAC: Participated in the construction of linkage maps. MTC, CP and IA: Designed experiments and coordinated projects. All authors contributed to writing the article and approved the final manuscript.

## Supplementary Material

Additional file 1 **Sequence of contigs and genes containing the analyzed SNPs.** Nucleotide sequences in fasta format of contigs and genes containing the polymorphic SNPs identified in the mapping progenies. All sequences are the consensus sequence obtained from different individuals except for genes *LP3-1* and *LP3-3* whit sequences obtained for one individual. Click here for file

Additional file 2 **Standard error of recombination frequency.** Representation of the standard error of recombination frequency for markers segregating 1:1 in the F_1_ mapping populations C14xC15 (N=106) and C15xC14 (N=55). Standard error calculated following Ritter et al. [99]. Click here for file

Additional file 3 **Polymorphic SNPs.** List of the polymorphic SNPs in mapping progenies and associated features. Click here for file

Additional file 4 **Distorted markers.** Number of total (assigned, ungrouped and excluded) markers showing distortion in their segregation rates at p ≤ 0.01 per LG obtained for C14 and C15 linkage maps constructed by merging two reciprocal crosses: C14xC15 and C15xC14. Click here for file

Additional file 5 **BLAST analysis of SNP-sequences.** Results of BLAST-N search in pine databases of the Gene Index Project and GeneBank for mapped SNPs. Click here for file

Additional file 6 **GO annotation for mapped SNPs.** Sequence distribution of GO terms for molecular function. Click here for file

## References

[B1] Blanco E Casado MA Costa M Escribano R García-Anton M Génova M Gómez-Manzaneque A Gómez-Manzaneque F Moreno JC Morla C Regato P Sainz H Los bosques ibéricos. Una interpretación geobotánica 2005 4 Barcelona: Planeta

[B2] Fernández M Gil L Pardos JA Effects of water supply on gas exchange in Pinus pinaster Ait. provenances during their first growing season Ann For Sci 2000 57 9 16 10.1051/forest:2000107

[B3] Sánchez-Gómez D Majada J Alía R Feito I Aranda I Intraspecific variation in growth and allocation patterns in seedlings of Pinus pinaster Ait. submitted to contrasting watering regimes: can water availability explain regional variation? Ann For Sci 2010 67 505 10.1051/forest/2010007

[B4] Aranda I Alía R Ortega U Dantas AK Majada J Intra-specific variability in biomass partitioning and carbon isotopic discrimination under moderate drought stress in seedlings from four Pinus pinaster populations Tree Genet Genomes 2010 6 169 178 10.1007/s11295-009-0238-5

[B5] Mariette S Chagné D Lézier C Pastuszka P Raffin A Plomion C Kremer A Genetic diversity within and among Pinus pinaster populations: comparison between AFLP and microsatellite markers Heredity 2001 86 469 479 10.1046/j.1365-2540.2001.00852.x 11520347

[B6] Ribeiro M Mariette S Vendramin G Szmidt A Plomion C Kremer A Comparison of genetic diversity estimates within and among populations of maritime pine using chloroplast simple-sequence repeat and amplified fragment length polymorphism data Mol Ecol 2002 11 869 877 10.1046/j.1365-294X.2002.01490.x 11975703

[B7] González-Martínez SC Mariette S Ribeiro MM Burban C Raffin A Chambel MR Ribeiro CAM Aguiar A Plomion C Alía R Gil L Vendramin GG Kremer A Genetic resources in maritime pine (Pinus pinaster Aiton): molecular and quantitative measures of genetic variation and differentation among maternal lineages For Ecol Manag 2004 197 103 115 10.1016/j.foreco.2004.05.008

[B8] Eveno E Collada C Guevara MA Léger V Soto A Díaz L Léger P González-Martínez SC Cervera MT Plomion C Garnier-Géré P Contrasting patterns of selection at Pinus pinaster Ait. drought stress candidate genes as revealed by genetic differentiation analyses Mol Biol Evol 2008 25 417 437 10.1093/molbev/msm272 18065486

[B9] Vendramin G Anzidei M Madaghiele A Bucci G Distribution of genetic diversity in Pinus pinaster Ait. as revealed by chloroplast microsatellites Theor Appl Genet 1998 97 456 463 10.1007/s001220050917

[B10] Bucci G González-Martínez SC Le Provost G Plomion C Ribeiro MM Sebastiani F Alía R Vendramin GG Range-wide phylogeography and gene zones in Pinus pinaster Ait. revealed by chloroplast microsatellite markers Molec Ecol 2007 16 2137 2153 10.1111/j.1365-294X.2007.03275.x 17498237

[B11] González-Martínez SC Alía R Gil L Population genetic structure in a Mediterranean pine (Pinus pinaster Ait.): a comparison of allozyme markers and quantitative traits Heredity 2002 89 199 206 10.1038/sj.hdy.6800114 12209390

[B12] Farjon A A natural history of Conifers 2008 Portland: Timber Press, Inc.

[B13] Tadesse W Nanos N Aunon F Arrabal C Garcia C Gil L Alia R Pardos J Genetic improvement of resin yield from maritime pine in Spain Forest Chemicals Review 2001 111 11

[B14] Nanos N Tadesse W Montero G Gil L Alia R Modelling resin production distributions for Pinus Pinaster Ait using two probability functions Ann For Sci 2000 57 369 377 10.1080/000337900750013507

[B15] Mergen F Hoekstra P Echols RM Genetic control of oleoresin yield and viscosity in Slash Pine For Sci 1955 1 19 30

[B16] White TL Adams WT Neale D White TL, Adams WT, Neale D Tree Improvement Programs-Structure, Concepts and Importance Forest Genetics 2007 Oxfordshire: CABI Publishing 285 302

[B17] Arrabal C García-Vallejo MC Cadahia E Cortijo M de Simón BF Characterization of two chemotypes of Pinus pinaster by their terpene and acid patterns in needles Plant Syst Evol 2012 298 511 522 10.1007/s00606-011-0562-8

[B18] Tadesse W Nanos N Alia R Aunon F Gil L Evaluation of high resin yielders of Pinus pinaster Ait International Journal of Forest Genetics 2001 8 271 278

[B19] Arrabal C Cortijo M De Simon BF García Vallejo MC Cadahía E Differentiation among five Spanish Pinus pinaster provenances based on its oleoresin terpenic composition Biochem Syst Ecol 2005 33 1007 1016 10.1016/j.bse.2005.03.003

[B20] Gerber S Lascoux M Kremer A Relation between protein markers and quantitative traits in maritime pine (Pinus pinaster AIT.) Silvae Genet 1997 46 286 291

[B21] Pelgas B Bousquet J Meirmans P Ritland K Isabel N QTL mapping in white spruce: gene maps and genomic regions underlying adaptive traits across pedigrees, years and environments BMC Genomics 2011 12 145 10.1186/1471-2164-12-145 21392393 PMC3068112

[B22] Devey ME Carson SD Nolan ME Matheson AC Te Riini C Hohepa J QTL associations for density and diameter in Pinus radiata and the potential for marker-aided selection Theor Appl Genet 2004 108 516 524 10.1007/s00122-003-1446-2 14657985

[B23] Plomion C Yani A Marpeau A Genetic determinism of delta 3-carene in maritime pine using RAPD markers Genome 1996 39 1123 1127 10.1139/g96-141 18469960

[B24] Pelgas B Bousquet J Beauseigle S Isabel N A composite linkage map from two crosses for the species complex Picea mariana× Picea rubens and analysis of synteny with other Pinaceae Theor Appl Genet 2005 111 1466 1488 10.1007/s00122-005-0068-2 16215729

[B25] Krutovsky KV Troggio M Brown GR Jermstad KD Neale DB Comparative mapping in the Pinaceae Genetics 2004 168 447 461 10.1534/genetics.104.028381 15454556 PMC1448108

[B26] Cervera MT Plomion C Malpica C Jain SM, Minocha SC Molecular markers and genome mapping in woody plants Molecular Biology of Woody Plants 2000 1 Dordrecht: Kluwer Academic Publishers 375 394

[B27] Devey ME Sewell MM Uren TL Neale DB Comparative mapping in loblolly and radiata pine using RFLP and microsatellite markers Theor Appl Genet 1999 99 656 662 10.1007/s001220051281 22665202

[B28] Vos P Hogers R Bleeker M Reijans M Lee T Hornes M Friters A Pot J Paleman J Kuiper M AFLP: a new technique for DNA fingerprinting Nucleic Acids Res 1995 23 4407 10.1093/nar/23.21.4407 7501463 PMC307397

[B29] Williams JGK Kubelik AR Livak KJ Rafalski JA Tingey SV DNA polymorphisms amplified by arbitrary primers are useful as genetic markers Nucleic Acids Res 1990 18 6531 6535 10.1093/nar/18.22.6531 1979162 PMC332606

[B30] Gosselin I Zhou Y Bousquet J Isabel N Megagametophyte-derived linkage maps of white spruce (Picea glauca) based on RAPD, SCAR and ESTP markers Theor Appl Genet 2002 104 987 997 10.1007/s00122-001-0823-y 12582604

[B31] Ritland K Krutovsky KV Tsumura Y Pelgas B Isabel N Bousquet J Plomion C, Bousquet J Genetic mapping in conifers Genetics, Genomics and Breeding of Conifers 2011 Edenbridge: Science Publishers 196 238

[B32] Remington D Whetten R Liu BH O'malley D Construction of an AFLP genetic map with nearly complete genome coverage in Pinus taeda Theor Appl Genet 1999 98 1279 1292 10.1007/s001220051194 12238515

[B33] Paglia G Morgante M PCR-based multiplex DNA fingerprinting techniques for the analysis of conifer genomes Mol Breed 1998 4 173 177 10.1023/A:1009637608702

[B34] González-Martínez SC Krutovsky KV Neale DB Forest-tree population genomics and adaptive evolution New Phytol 2006 170 227 238 10.1111/j.1469-8137.2006.01686.x 16608450

[B35] Komulainen P Brown GR Mikkonen M Karhu A Garcia-Gil MR O'Malley D Lee B Neale DB Savolainen O Comparing EST-based genetic maps between Pinus sylvestris and Pinus taeda Theor Appl Genet 2003 107 667 678 10.1007/s00122-003-1312-2 12827250

[B36] Tani N Takahashi T Iwata H Mukai Y Ujino-Ihara T Matsumoto A Yoshimura K Yoshimaru H Murai M Nagasaka K Tsumura Y A consensus linkage map for Sugi (Cryptomeria japonica) from two pedigrees, based on microsatellites and Expressed Sequence Tags Genetics 2003 165 1551 1568 14668402 10.1093/genetics/165.3.1551 PMC1462850

[B37] Plomion C Hurme P Frigerio J Ridolfi M Pot D Pionneau C Avila C Gallardo F David H Neutelings G Developing SSCP markers in two Pinus species Mol Breed 1999 5 21 31 10.1023/A:1009635226412

[B38] Echt C Saha S Krutovsky K Wimalanathan K Erpelding J Liang C An annotated genetic map of loblolly pine based on microsatellite and cDNA markers BMC Genet 2011 12 17 21269494 10.1186/1471-2156-12-17 PMC3038140

[B39] Neale DB Genomics to tree breeding and forest health Curr Opin Genet Dev 2007 17 539 544 10.1016/j.gde.2007.10.002 18060764

[B40] Cho RJ Mindrinos M Richards DR Sapolsky RJ Anderson M Drenkard E Dewdney J Reuber TL Stammers M Federspiel N Genome-wide mapping with biallelic markers in Arabidopsis thaliana Nat Genet 1999 23 203 207 10.1038/13833 10508518

[B41] Lepoittevin C Frigerio JM Garnier-Géré P Salin F Cervera MT Vornam B Harvengt L Plomion C In Vitro vs In Silico Detected SNPs for the Development of a Genotyping Array: What Can We Learn from a Non-Model Species? PLoS ONE 2010 5 e11034 10.1371/journal.pone.0011034 20543950 PMC2882948

[B42] Pavy N Parsons L Paule C MacKay J Bousquet J Automated SNP detection from a large collection of white spruce expressed sequences: contributing factors and approaches for the categorization of SNPs BMC Genomics 2006 7 174 10.1186/1471-2164-7-174 16824208 PMC1557672

[B43] Eckert AJ Pande B Ersoz ES Wright MH Rashbrook VK Nicolet CM Neale DB High-throughput genotyping and mapping of single nucleotide polymorphisms in loblolly pine (Pinus taeda L.) Tree Genet Genomes 2009 5 225 234 10.1007/s11295-008-0183-8

[B44] Chancerel E Lepoittevin C Le Provost G Lin Y-C Jaramillo-Correa J Eckert A Wegrzyn J Zelenika D Boland A Frigerio J-M Chaumeil P Garnier-Gere P Boury C Grivet D Gonzalez-Martinez S Rouze P Van de Peer Y Neale D Cervera M Kremer A Plomion C Development and implementation of a highly-multiplexed SNP array for genetic mapping in maritime pine and comparative mapping with loblolly pine BMC Genomics 2011 12 368 10.1186/1471-2164-12-368 21767361 PMC3146957

[B45] Pavy N Pelgas B Beauseigle S Blais S Gagnon F Gosselin I Lamothe M Isabel N Bousquet J Enhancing genetic mapping of complex genomes through the design of highly-multiplexed SNP arrays: application to the large and unsequenced genomes of white spruce and black spruce BMC Genomics 2008 9 21 10.1186/1471-2164-9-21 18205909 PMC2246113

[B46] Moriguchi Y Ujino-Ihara T Futamura N Saito M Ueno S Matsumoto A Tani N Taira H Shinohara K Tsumura Y The construction of a high-density linkage map for identifying SNP markers that are tighly linked to a nuclear-recessive major gene for male sterility in Cryptomeria japonica D.Don BMC Genomics 2012 19 95 22424262 10.1186/1471-2164-13-95 PMC3386010

[B47] Guevara MA Cervera MT Soto A Collada C Plomion C Savolainen O Neale DB González-Martínez SC Genomics applied to the study of adaptation in pine species Investigacion Agraria: Sistemas y Recursos Forestales 2005 14 292 306

[B48] Elsik C Williams C Retroelements contribute to the excess low-copy-number DNA in pine Mol Gen Genet 2000 264 47 55 10.1007/s004380000279 11016832

[B49] Chagné D Lalanne C Madur D Kumar S Frigério JM Krier C Decroocq S Savouré A Bou-Dagher-Kharrat M Bertocchi E Brach J Plomion C A high density genetic map of maritime pine based on AFLPs Ann For Sci 2002 59 627 636 10.1051/forest:2002048

[B50] Grotkopp E Rejmánek M Sanderson MJ Rost TL Evolution of genome size in pines (Pinus) and its life-history correlates: supertree analyses Evolution 2004 58 1705 1729 15446425 10.1111/j.0014-3820.2004.tb00456.x

[B51] Bahrman N Damerval C Linkage relationships of loci controlling protein amounts in maritime pine (Pinus pinaster Ait) Heredity 1989 63 267 274 10.1038/hdy.1989.99

[B52] Gerber S Rodolphe F Bahrman N Baradat P Seed-protein variation in maritime pine (Pinus pinaster Ait) revealed by two-dimensional electrophoresis: genetic determinism and construction of a linkage map Theor Appl Genet 1993 85 521 528 10.1007/BF00220908 24195924

[B53] Plomion C Costa P Bahrman N Genetic analysis of needle protein in Maritime pine. Mapping dominant and codominant protein markers assayed on diploid tissue, in a haploid-base genetic map Silvae Genet 1997 46 161 165

[B54] Costa P Pot D Dubos C Frigerio JM Pionneau C Bodenes C Bertocchi E Cervera M-T Remington DL Plomion C A genetic map of Maritime pine based on AFLP, RAPD and protein markers Theor Appl Genet 2000 100 39 48 10.1007/s001220050006

[B55] Plomion C Bahrman N Durel CE O'Malley DM Genomic mapping in Pinus pinaster (maritime pine) using RAPD and protein markers Heredity 1995 74 661 668 10.1038/hdy.1995.90

[B56] Plomion C O'Malley D Durel C-E Genomic analysis in maritime pine (Pinus pinaster) comparison of two RAPD maps using selfed and open-pollinated seeds of the same individual Theor Appl Genet 1995 90 1028 1034 10.1007/BF00222917 24173058

[B57] Plomion C O'Malley D Recombination rate differences for pollen parents and seed parents in pine Heredity 1996 77 341 350 10.1038/hdy.1996.152

[B58] Ritter E Aragones A Markussen T Achere V Espinel S Fladung M Wrobel S Faivre-Rampant P Jeandroz S Favre JM Towards construction of an ultra high density linkage map for Pinus pinaster Annals of Forest Sciences 2002 59 637 643 10.1051/forest:2002049

[B59] Mariette S Changné D Decroocq S Vendramin GG Lalanne C Madur D Plomion C Microsatellite markers for Pinus pinaster Ait Ann For Sci 2001 58 203 206

[B60] Chagné D Chaumeil P Ramboer A Collada C Guevara MA Cervera MT Vendramin GG Garcia V Frigerio J-M Echt C Richardson T Plomion C Cross-species transferability and mapping of genomic and cDNA SSRs in pines Theor Appl Genet 2004 109 1204 1214 10.1007/s00122-004-1683-z 15448894

[B61] Chagné D Brown GR Lalanne C Madur D Pot D Neale D Plomion C Comparative genome and QTL mapping between maritime and loblolly pines Mol Breed 2003 12 185 195 10.1023/A:1026318327911

[B62] Brendel O Pot D Plomion C Rozenberg P Guehl JM Genetic parameters and QTL analysis of delta C-13 and ring width in maritime pine Plant Cell Environ 2002 25 945 953 10.1046/j.1365-3040.2002.00872.x

[B63] Markussen T Fladung M Achere V Favre JM Faivre-Rampant P Aragones A DA Silva Pérez D Havengt L Ritter E Identification of QTLs controlling growth, chemical and physical wood property traits in Pinus pinaster, Ait Silvae Genet 2003 52 8 15

[B64] Pot D Rodrigues J Rozenberg P Chantre G Tibbits J Cahalan C Pichavant F Plomion C QTLs and candidate genes for wood properties in maritime pine (Pinus pinaster Ait.) Tree Genet Genomes 2006 2 10 24 10.1007/s11295-005-0026-9

[B65] Dellaporta SL Wood J Hicks JB A plant DNA minipreparation: version II Plant Mol Biol Report 1983 1 19 21 10.1007/BF02712670

[B66] Guevara MA Chagné D Almeida H Byrnes M Collada C Favre JM Harvengt L Jeandroz S Orazio C Plomion C Ramboer A Rocheta M Sebastiani F Soto A Vendramin GG Cervera MT Isolation and characterization of nuclear microsatellite loci in Pinus pinaster Ait Mol Ecol Notes 2005 5 57 59 10.1111/j.1471-8286.2004.00830.x

[B67] Elsik CG Williams CG Low copy microsatellite recovery from a conifer genome Theor Appl Genet 2001 103 1189 1195 10.1007/s001220100725

[B68] Auckland L Conifer microsatellite handbook 2002 Raleigh: Texas A & M University

[B69] González-Martínez SC Robledo-Arnuncio JJ Collada C Díaz A Williams CG Alía R Cervera MT Cross -amplification and sequence variation of microsatellite loci in Eurasian hard pines Theor Appl Genet 2004 109 103 111 10.1007/s00122-004-1596-x 14985972

[B70] Till BJ Reynolds SH Greene EA Codomo CA Enns LC Johnson JE Burtner C Odden AR Young K Taylor NE Henikoff JG Comai L Henikoff S Large-Scale Discovery of Induced Point Mutations With High-Throughput TILLING Genome Res 2003 13 524 530 10.1101/gr.977903 12618384 PMC430291

[B71] Rungis D Hamberger B Bérubé Y Wilkin J Bohlmann J Ritland K Efficient genetic mapping of single nucleotide polymorphims based upon DNA mismatch digestion Mol Breed 2005 16 261 270 10.1007/s11032-005-3424-7

[B72] Brown G Kadel E III Bassoni D Kiehne K Temesgen B Van Buijtenen J Sewell M Marshall K Neale D Anchored reference loci in loblolly pine (Pinus taeda L.) for integrating pine genomics Genetics 2001 159 799 809 11606554 10.1093/genetics/159.2.799 PMC1461821

[B73] Temesgen B Brown GR Harry DE Kinlaw CS Sewell MM Neale DB Genetic mapping of expressed sequence tag polymorphism (ESTP) markers in loblolly pine (Pinus taeda L.) Theor Appl Genet 2001 102 664 675 10.1007/s001220051695

[B74] Cervera M Gusmao J Steenackers M Peleman J Storme V Vanden Broeck A Van Montagu M Boerjan W Identification of AFLP molecular markers for resistance against Melampsora larici-populina in Populus Theor Appl Genet 1996 93 733 737 10.1007/BF00224069 24162401

[B75] Schmidt A Doudrick R Heslop-Harrison J Schmidt T The contribution of short repeats of low sequence complexity to large conifer genomes Theor Appl Genet 2000 101 7 14 10.1007/s001220051442

[B76] Grivet D Sebastiani F Alía R Bataillon T Torre S Zabal-Aguirre M Vendramin GG González-Martínez SC Molecular footprints of local adaptation in two Mediterranean conifers Mol Biol Evol 2011 28 101 10.1093/molbev/msq190 20656795

[B77] Grattapaglia D Sederoff R Genetic linkage maps of Eucalyptus grandis and Eucalyptus urophylla using a pseudo-testcross mapping strategy and RAPD markers Genetics 1994 137 1121 1137 7982566 10.1093/genetics/137.4.1121 PMC1206059

[B78] Van Ooijen JW Kiazma BV Joinmap 4, software for the calculation of genetic maps in experimental populations 2006 Wageningen

[B79] Kosambi D The estimation of map distances from recombination values Annals Eugen 1944 12 172 175

[B80] Hulbert S Ilott T Legg E Lincoln S Lander E Michelmore R Genetic analysis of the fungus, Bremia lactucae, using restriction fragment length polymorphisms Genetics 1988 120 947 2906309 10.1093/genetics/120.4.947 PMC1203586

[B81] Chakravarti A Lasher LK Reefer JE A maximum likelihood method for estimating genome length using genetic linkage data Genetics 1991 128 175 182 2060775 10.1093/genetics/128.1.175 PMC1204446

[B82] Cervera M-T Storme V Ivens B Gusmão J Liu BH Hostyn V Van Slycken J Van Montagu M Boerjan W Dense Genetic Linkage Maps of Three Populus Species (Populus deltoides, P. nigra and P. trichocarpa) Based on AFLP and Microsatellite Markers Genetics 2001 158 787 809 11404342 10.1093/genetics/158.2.787 PMC1461694

[B83] The Gene Index Project http://compbio.dfci.harvard.edu/tgi/cgi-bin/tgi/Blast/index.cgi

[B84] Gene Bankhttp://www.ncbi.nlm.nih.gov/genbank/

[B85] Conesa A Götz S García-Gomez JM Terol J Talon M Robles M Blast2GO:a universal tool for annotation, visualization and analysis in functional genomics research Bioinformatics 2005 21 3674 3676 10.1093/bioinformatics/bti610 16081474

[B86] Pot D Chantre G Rozenberg P Rodrigues JC Jones GL Pereira H Hannrup B Cahalan C Plomion C Genetic control of pulp and timber properties in maritime pine (Pinus pinaster Ait.) Ann For Sci 2002 59 563 575 10.1051/forest:2002042

[B87] Scotti I Burelli A Cattonaro F Chagné D Fuller J Hedley PE Jansson G Lalanne C Madur D Neale D Plomion C Powell W Troggio M Morgante M Analysis of the distribution of marker classes in a genetic linkage map: a case study in Norway spruce (Picea abies Karst) Tree Genet Genomes 2005 1 93 102 10.1007/s11295-005-0012-2

[B88] Gerber S Rodolphe F An estimation of the genome length of maritime pine (Pinus pinaster Ait.) Theor Appl Genet 1994 88 289 292 10.1007/BF00223634 24186008

[B89] Sax K Sax HJ Chromosome number and morphology in the conifers J Arnold Arbor 1933 14 356 375

[B90] González-Martínez S Estructura poblacional y flujo genético de Pinus pinaster Aiton en el noroeste de la Península Ibérica. PhD Thesis 2001 Universidad Politécnica de Madrid, ETSIM

[B91] Nordborg M Hu TT Ishino Y Jhaveri J Toomajian C Zheng H Bakker E Calabrese P Gladstone J Goyal R Jakobsson M Kim S Morozov Y Padhukasahasram B Plagnol V Rosenberg NA Shah C Wall JD Wang J Zhao K Kalbfleisch T Schulz V Kreitman M Bergelson J The pattern of polymorphism in Arabidopsis thaliana PLoS Biol 2005 3 e196 10.1371/journal.pbio.0030196 15907155 PMC1135296

[B92] Yin TM Wang XR Andersson B Lerceteau-Köhler E Nearly complete genetic maps of Pinus sylvestris L.(Scots pine) constructed by AFLP marker analysis in a full-sib family Theor Appl Genet 2003 106 1075 1083 12671756 10.1007/s00122-003-1194-3

[B93] Kubisiak TL Nelson CD Nance W Stine M RAPD linkage mapping in a longleaf pine x slash pine F 1 family Theor Appl Genet 1995 90 1119 1127 10.1007/BF00222931 24173072

[B94] Echt C Nelson C Linkage mapping and genome length in eastern white pine (Pinus strobus L.) Theor Appl Genet 1997 94 1031 1037 10.1007/s001220050511

[B95] Yin T DiFazio SP Gunter LE Riemenschneider D Tuskan GA Large-scale heterospecific segregation distortion in Populus revealed by a dense genetic map Theor Appl Genet 2004 109 451 463 15168022 10.1007/s00122-004-1653-5

[B96] InterPro protein sequence analysis & classification http://www.ebi.ac.uk/interpro/

[B97] Farquhar GD Richards RA Isotopic composition of plant carbon correlates with water-use efficiency of wheat genotypes Aust J Plant Physiol 1984 11 539 552 10.1071/PP9840539

[B98] Boerjan W Ralph J Baucher M Lignin biosynthesis Annu Rev Plant Biol 2003 54 519 546 10.1146/annurev.arplant.54.031902.134938 14503002

[B99] Ritter E Gebhardt C Salamini F Estimation of recombination frequencies and construction of RFLP linkage maps in Plants from crosses between heterozygous parents Genetics 1990 125 645 654 1974227 10.1093/genetics/125.3.645 PMC1204090

