# Supplementary figures and images for: Annotated genetic linkage maps of Pinus pinaster Ait. from a Central Spain population using microsatellite and gene based markers
Source: BMC Genomics. 2012 Oct 4;13:527. doi: 10.1186/1471-2164-13-527 (PMC3534022; doi:10.1186/1471-2164-13-527)

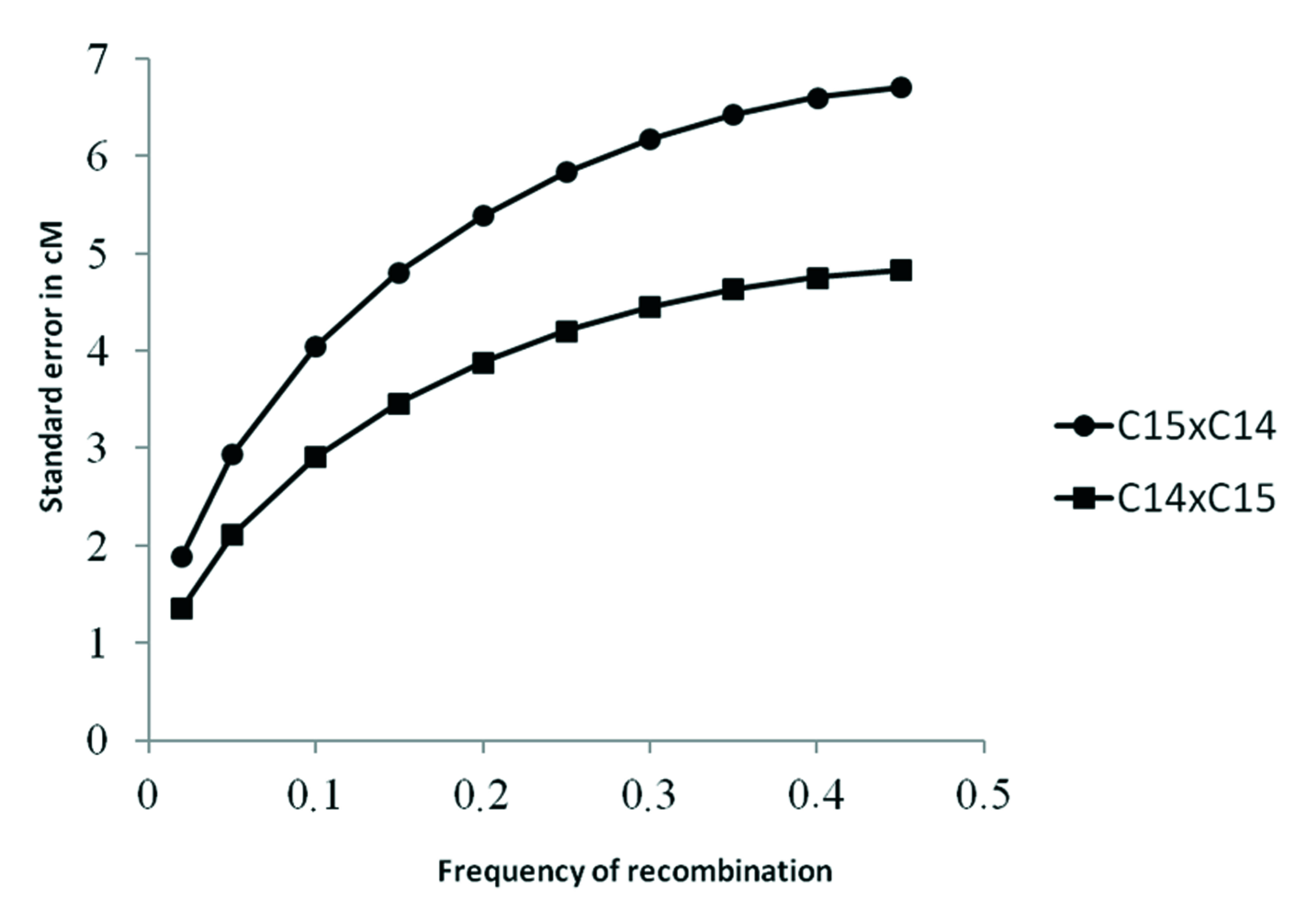

Supplement: Additional file 2 — Standard error of recombination frequency. Representation of the standard error of recombination frequency for markers segregating 1:1 in the F1 mapping populations C14xC15 (N=106) and C15xC14 (N=55). Standard error calculated following Ritter et al. [99]. [file 1471-2164-13-527-S2.jpeg]

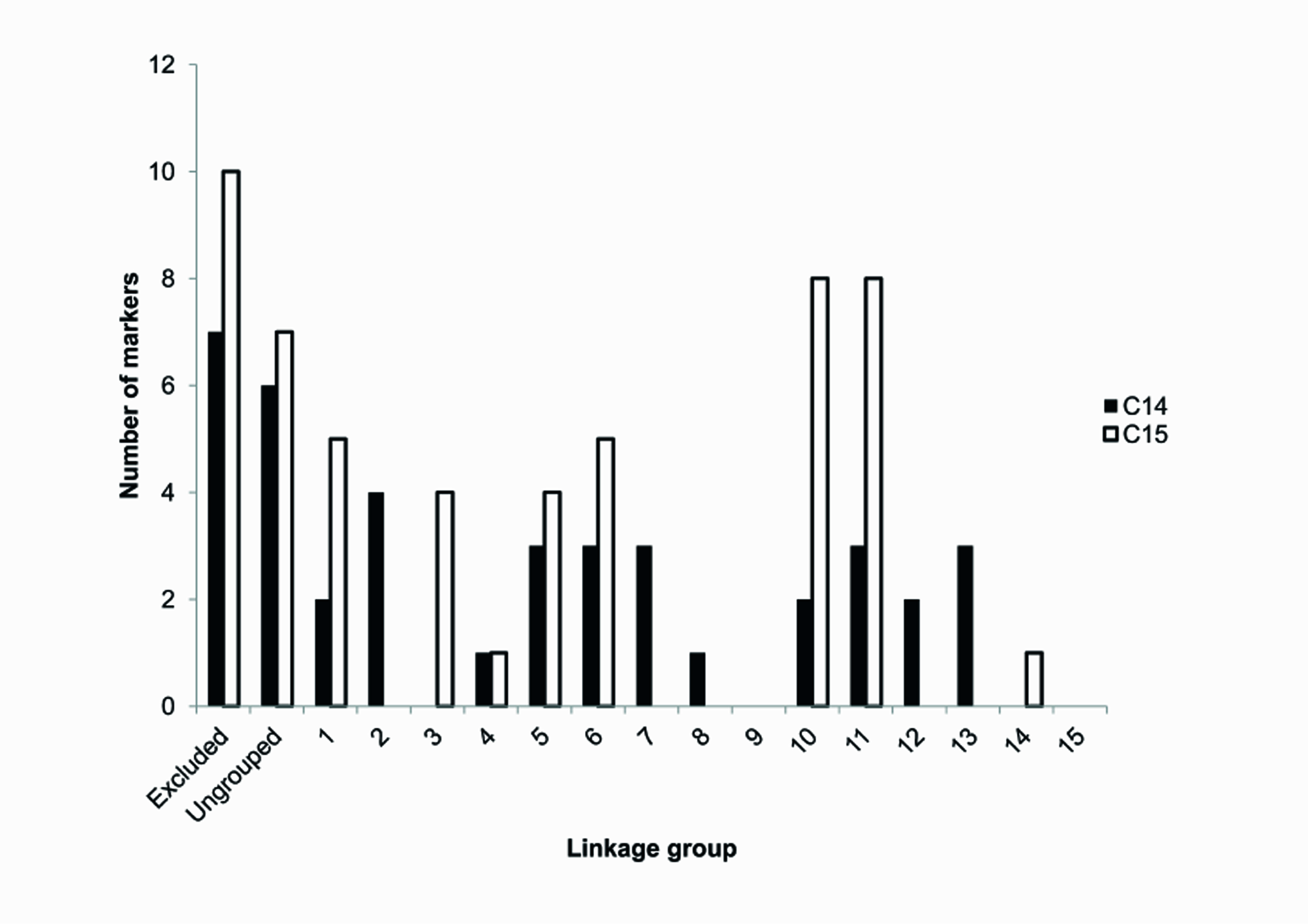

Supplement: Additional file 4 — Distorted markers. Number of total (assigned, ungrouped and excluded) markers showing distortion in their segregation rates at p ≤ 0.01 per LG obtained for C14 and C15 linkage maps constructed by merging two reciprocal crosses: C14xC15 and C15xC14. [file 1471-2164-13-527-S4.jpeg]

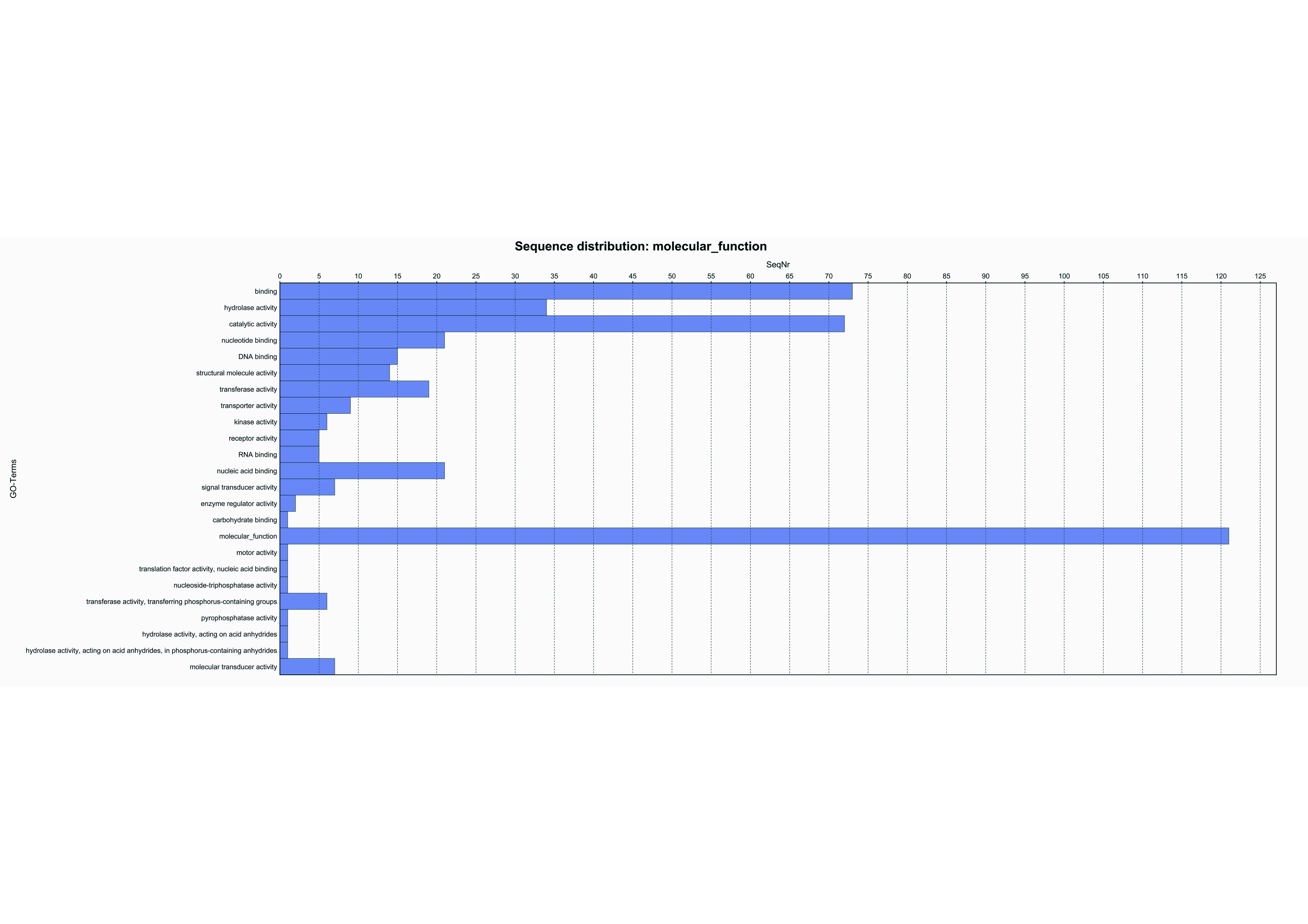

Supplement: Additional file 6 — GO annotation for mapped SNPs. Sequence distribution of GO terms for molecular function. [file 1471-2164-13-527-S6.jpeg]
